# Plasma Amino Acid Signatures Associated with Disease Progression and Hypertension in Autosomal Dominant Polycystic Kidney Disease: A Targeted Metabolomics and Machine Learning Approach

**DOI:** 10.3390/jcm15145340

**Published:** 2026-07-08

**Authors:** Leila Kianmehr, Gözde Ertürk Zararsız, Ahu Cephe, Nida Sofu, Alparslan Demiray, Salih Güntuğ Özaytürk, Halef Okan Doğan, İsmail Koçyiğit, Gökmen Zararsız

**Affiliations:** 1 Department of Biostatistics, Faculty of Medicine, Erciyes University, Kayseri 38039, Türkiye; l_kianmehr@erciyes.edu.tr (L.K.); gokmenzararsiz@erciyes.edu.tr (G.Z.); 2 Institutional Data Management and Analytics Coordination Unit, Erciyes University, Kayseri 38039, Türkiye; ahucephe@erciyes.edu.tr; 3 Department of Bioinformatics and Systems Biology, Institute of Health Sciences, Erciyes University, Kayseri 38039, Türkiye; nidasofu@gmail.com; 4 Division of Nephrology, Faculty of Medicine, Erciyes University, Kayseri 38039, Türkiye; alparslan1025@gmail.com (A.D.); salih.ozayturk@erciyes.edu.tr (S.G.Ö.); ikocyigit@erciyes.edu.tr (İ.K.); 5 Hematainer Biotechnology and Health Products Inc., Erciyes Teknopark, Kayseri 38039, Türkiye; hodogan@cumhuriyet.edu.tr; 6 Department of Biochemistry, School of Medicine, Sivas Cumhuriyet University, Sivas 58140, Türkiye

**Keywords:** autosomal dominant polycystic kidney disease (ADPKD), amino acids, BCAAs, targeted metabolomics, LC-MS/MS, disease progression, machine learning

## Abstract

**Background:** Autosomal dominant polycystic kidney disease (ADPKD) is a clinically heterogeneous disorder often leading to end-stage renal disease (ESRD). Prognostication of disease progression remains a major clinical challenge. This study aimed to identify plasma amino acid signatures associated with ADPKD progression and hypertension. **Methods:** We conducted targeted metabolomic analysis (LC-MS/MS) to quantify 38 plasma amino acids in 203 ADPKD patients, stratified by disease progression (rapid vs. slow) and hypertension status. Support Vector Machine (SVM) models were developed to predict outcomes using clinical data, amino acid profiles, and combined datasets. **Results:** Our findings revealed that specific amino acid signatures, including valine, glutamic acid, homocitrulline, and methylhistidines, were significantly elevated in both rapid progression and hypertensive groups. Isoleucine and citrulline were elevated only in rapid progressors. Phenylalanine, leucine, asparagine, and arginine were elevated in hypertensive patients. Machine learning analysis showed that integrating clinical and metabolic data modestly improved prediction for progression and hypertension. Proteinuria, glomerular filtration rate (GFR), and uric acid were the top clinical predictors; however, adding arginine, isoleucine, and 3-methylhistidine further enhanced prediction accuracy. Pathway analysis showed shared dysregulation in arginine biosynthesis and branched-chain amino acid (BCAA) metabolism. Specific amino acids were positively correlated with creatinine and uric acid and negatively correlated with GFR, and elevated levels of these metabolites were associated with increased mortality risk in survival analysis. **Conclusions:** Our results suggest that these plasma amino acid signatures, when combined with clinical markers, may serve as potential biomarkers for early risk stratification and precision prediction of ADPKD progression and hypertension.

## 1. Introduction

Autosomal dominant polycystic kidney disease (ADPKD) is the most common inherited kidney disorder, characterized by the formation and progressive enlargement of fluid-filled cysts. These structural changes drive massive kidney growth, declining renal function, and ultimately end-stage renal disease (ESRD) [1,2]. The clinical course of ADPKD is highly heterogeneous, influenced by genetic mutations (particularly *PKD1* and *PKD2*) and clinical comorbidities such as hypertension, which precedes the decline in glomerular filtration rate (GFR), making prognostication of disease progression in patients a persistent and crucial clinical challenge [3,4].

Current risk stratification in ADPKD relies on imaging-based techniques such as Mayo Imaging Classification (MIC) and GFR, yet these metrics fail to detect early disease progression [5,6]. Consequently, there is a critical need to leverage the growing understanding of ADPKD pathogenesis, specifically metabolic reprogramming and dysregulated amino acid metabolism, to identify novel biomarkers, such as plasma metabolites that can reflect dynamic pathophysiological changes to detect disease progression earlier and monitor therapeutic response.

The kidney acts as a central regulator of systemic amino acid homeostasis, overseeing filtration, reabsorption, and inter-organ exchange. In renal disease such as ADPKD, these pathways are disrupted, contributing to systemic complications, including metabolic acidosis, sarcopenia, inflammation, and oxidative stress. Beyond these, metabolic reprogramming is a hallmark of ADPKD pathogenesis [7,8]. Amino acids are central for this metabolic rewiring, serving not only as substrates for energy production but also as key regulators of signaling pathways that drive cyst expansion [9]. Dysregulation of specific amino acids, including arginine, branched-chain amino acids (BCAAs), and phenylalanine, has been observed in CKD. Assessing targeted amino acids in a longitudinal cohort of ADPKD patients provides potential biomarkers for monitoring disease progression [10,11].

Previous metabolomic studies in ADPKD have examined cross-sectional associations between plasma metabolite concentrations and renal function [7,12,13]. Nevertheless, the majority of these prior investigations utilized untargeted approaches, analyzed relatively small cohorts, or focused on descriptive metabolic differences without integration with validated clinical risk stratification models such as MIC. Our group has also recently shared early findings on changes in metabolism, including in amino acid pathways, using untargeted metabolomics [14]. To validate these findings and translate them into clinical utility, a quantitative targeted analysis of specific amino acid profiles linked to established progression markers and clinical outcomes is required.

In this study, we conducted a targeted metabolomic analysis of 38 plasma amino acids in a large cohort of ADPKD patients. We aimed to characterize the amino acid signatures associated with rapid disease progression and hypertension. Additionally, we implemented an advanced machine learning model to evaluate whether integrating amino acid profiles with standard clinical parameters could improve the accuracy of predictions of disease severity and patient outcomes.

## 2. Materials and Methods

### 2.1. Study Design and Participants

This single-center, observational, ambidirectional (retrospective and prospective) cohort study was conducted at the Nephrology Clinic of Erciyes University, Faculty of Medicine, between 2017 and 2024. The study comprised 203 patients diagnosed with ADPKD, who were monitored for a period of up to seven years. To our knowledge, this represents the largest targeted metabolomics study of ADPKD in Türkiye to date. This cohort, located at the intersection of Europe and the Middle East, provides a genetically and environmentally diverse patient population, hence enhancing the global relevance of the findings.

The cohort comprised adults between the ages of 30 and 70 years. The clinical diagnosis of ADPKD was established based on the Pei-Ravine unified diagnostic criteria, as endorsed by the KDIGO 2025 guidelines. This required either specific age-dependent cyst counts on ultrasound imaging or the identification of a pathogenic mutation in *PKD1* or *PKD2* via genetic testing [15,16]. Participants were either newly diagnosed or undergoing longitudinal follow-up at the time of enrollment. The study protocol was approved by the National Research Ethics Committee (2022/587) and conducted in strict compliance with the Declaration of Helsinki. All participants provided written informed consent.

#### Patient Stratification

To assess disease severity and phenotypic variability, patients were stratified according to two key clinical parameters: disease progression and hypertension status. Using the MIC, patients were stratified into predicted slow progression (MIC 1A and 1B) and rapid progression (1C–1E). Patients were categorized as hypertensive or non-hypertensive based on clinical history and blood pressure measurements.

### 2.2. Clinical and Biochemical Measurements

Baseline demographic data, including age, gender, body mass index (BMI), and *PKD* mutation status, were recorded (Table 1). A comprehensive panel of biochemical and hematological parameters was determined using standard laboratory methods. Plasma creatinine, uric acid, calcium, electrolytes (sodium, potassium), albumin, glucose, and lipid profile markers—including triglycerides (TG), total cholesterol (TC), high-density lipoprotein cholesterol (HDL), and low-density lipoprotein cholesterol (LDL)—were measured using colorimetric methods on a Roche Cobas c702 analyzer (Roche Diagnostics, Mannheim, Germany). GFR was calculated using the CKD-EPI formula. Hemoglobin was determined using a Sysmex XN 1000 hemogram analyzer (Sysmex Corporation, Kobe, Japan). Additionally, inflammation was assessed by measuring plasma C-reactive protein (CRP), and proteinuria was quantified from urine samples. Additionally, clinical parameters were monitored during the follow-up.

### 2.3. Sample Collection and Preparation

Blood plasma samples were collected in lithium heparin tubes within the first hour of hospital admission. Samples were centrifuged at 4 °C (10,000 rpm, 15 min), and plasma was aliquoted and stored at −80 °C. In order to conduct the analysis, 200 µL of plasma was mixed with 600 µL of methanol, incubated (5 min), and then placed at 4 °C (15 min) for protein precipitation. Subsequently, the samples were centrifuged at 13.000 rpm (15.000× *g*) and 4 °C (15 min). The supernatant was dried utilizing a vacuum concentrator for 12 h and reconstituted in 100 µL methanol (Thermo Fisher Scientific, Waltham, MA, USA). To remove any remaining insoluble debris, the reconstituted samples were centrifuged at 13.000 rpm (15.000× *g*) and 4 °C for 15 min before analysis.

### 2.4. LC-MS/MS Targeted Metabolomics

Quantification of 38 amino acids was performed using the Jasem Quantitative Amino Acids LC-MS/MS Kit (Altium International Lab. Cih. A.S., Istanbul, Türkiye). 50 µL of a sample was transferred into a tube using a pipette. Subsequently, 50 µL of the internal standard (IS) mixture solution was incorporated and stirred for 5 s at room temperature. Subsequently, 700 µL of reagent 1 (protein precipitation) was added to the vial, and the mixture was centrifuged at 3600× *g* for 5 min. The supernatant was aliquoted into a vial before injection into the LC-MS/MS apparatus.

The experiments were conducted using the Agilent high-performance liquid chromatography (HPLC) system (Agilent Technologies, Santa Clara, CA, USA), which comprises a 1290 high-speed pump (G7120A), a 1290 multisample injector (G7129A), and a 1260 multicolumn thermostat (G7116A), all connected to an Agilent 6470 triple quadrupole liquid chromatography-mass spectrometry (LC/MS) system (6470A, Agilent Technologies) featuring an electrospray ionization source. The amounts of free amino acids were measured using the CE-IVD approved and validated Jasem Quantitative Amino Acids LC-MS/MS Analysis Kit. Calibrators, quality controls, and plasma specimens were prepared according to the kit preparation protocol.

The technique for preparing calibration standards was as follows: fifty microliters of the calibrator were placed into a sample vial. Subsequently, 50 µL of the internal standard and 700 µL of reagent-1 were introduced into the vial. Following a 5 s vortex, the vial was positioned on an autosampler for injection into the LC-MS/MS system. Fifty microliters of the plasma specimens and quality controls were put into a glass tube. Subsequently, 50 µL of the internal standard and 700 µL of reagent-1 were added to the tube. After a five-second vortex, the tube was centrifuged at 3600× *g* for five minutes at ambient temperature. The supernatant was transferred to a vial before LC-MS/MS analysis. The chromatographic separation of underivatized free amino acids was accomplished utilizing the kit’s mobile phases, analytical column, and gradient elution protocol. The HPLC equipment was utilized to inject 3 µL of treated calibrators, controls, and samples into the analytical column maintained at 30 °C. Chromatographic separation was performed with Jasem’s mobile phases A and B with gradient elution at a flow rate of 0.7 mL/min. The HPLC elution proceeded as follows: the initial LC gradient of 22% A was maintained for one minute.

The gradient was raised incrementally to 78% B over 3.0 min and sustained for 0.5 min. The column was equilibrated at 22% A for three minutes. The overall duration was 7.5 min. Amino acids were detected in their entirety using positive ion multiple reaction monitoring (MRM) mode. The mass spectrometer parameters for the analytical procedure were drying gas temperature at 150 °C, drying gas flow at 10 L/min, nebulizer pressure at 40 psi, sheath gas temperature at 400 °C, sheath gas flow at 10 L/min, and capillary voltage at 2000 V. The MS/MS detections were achieved by product ion transitions produced by the collision-induced dissociation (CID) of the respective precursor ion. The MRM transitions of the amino acids and designated internal standards were observed at optimal fragmentation voltages (FV), which denoted the uniform value for each precursor/ion-product ion mass transition, and optimal collision energies (CE), which specified the particular value for each production in voltage (V) units. The quantification of analytes was performed using calibration curves based on the calibrators’ concentrations, accounting for matrix effects and process losses, and using the internal standard’s yields. Data acquisition, qualification, and quantification were conducted using the Agilent MassHunter software suite, specifically Acquisition (version 10.1), Agilent MassHunter Qualitative Analysis (version 10.0), and Agilent MassHunter Quantitative Analysis (version 10.0).

### 2.5. Statistical Analysis

Data distribution was assessed using histograms, Q-Q plots, and the Shapiro–Wilk test. Continuous variables were presented as mean ± standard deviation or median (25th–75th percentile), and categorical variables were summarized as *n* (%). Variables with a parametric distribution were compared using an independent two-sample *t*-test; otherwise, the Mann–Whitney *U* test was used. Categorical variables were compared using the Chi-square test. Amino acid concentrations were compared between disease progression groups and hypertension status groups. To control for false discovery rates in high-dimensional data, *p*-values were adjusted using the Benjamini–Hochberg procedure [17]. Pathway analyses were conducted using MetaboAnalyst 5.0 [18]. For survival analysis, optimal cut-off values for each amino acid were determined by maximizing the log-rank test statistic. The association of amino acids, categorized according to the determined cut-off values, with survival was investigated, and Kaplan–Meier plots illustrating survival differences were generated. The correlation between amino acids and clinical parameters was assessed using Spearman’s rank correlation analysis and visualized using correlation heatmaps. Additionally, hierarchical clustering was performed to visualize amino acid profiles across all samples.

### 2.6. Machine Learning Modeling

#### 2.6.1. Preprocessing and Feature Selection

Missing categorical variables were imputed using the Multivariate Imputation by Chained Equations (MICE) algorithm [19] and proportional odds logistic regression to preserve the ordinal structure of the variables. Missing values in continuous variables were imputed using the k-nearest neighbors (k-NN) method [20]. Subsequent to imputation, all continuous variables were standardized. Feature selection was performed using the Least Absolute Shrinkage and Selection Operator (LASSO) to reduce dimensionality and identify the most informative predictors before model construction [21]. Classification for disease progression and hypertension was implemented using a Support Vector Machine (SVM) with a polynomial kernel [22].

#### 2.6.2. Model Development and Evaluation

Machine learning-based classification models were developed to predict (i) disease progression (rapid vs. slow) and (ii) hypertension status (present vs. absent). Three different feature sets were evaluated for each outcome: clinical variables only, amino acid variables only, and combined clinical and amino acid variables. The SVM models were implemented with a polynomial degree of 1 and a scale parameter of 0.1. The regularization parameter (C) was set to 1 for disease progression models and 0.5 for hypertension models. Model performance was evaluated using repeated 5-fold cross-validation with 50 repetitions. Performance metrics included sensitivity (SENS), specificity (SPEC), balanced accuracy (BACC), Matthews correlation coefficient (MCC), F1-score, positive predictive value (PPV), and negative predictive value (NPV). Variable importance was assessed by the contribution of selected features in the final combined models. All statistical analyses and machine learning model development procedures were performed using R software version 4.3.1 (R Foundation for Statistical Computing, Vienna, Austria) [23], with statistical significance set at *p* < 0.05 (or adj. *p* < 0.05 where applicable).

## 3. Results

The final cohort consisted of 203 patients: 131 in the rapid-progression group and 72 in the slow-progression group. Regarding hypertension status, the cohort included 111 hypertensive and 92 non-hypertensive patients. The demographic and laboratory characteristics of these study groups are summarized in Table 1.

The sex distribution varied significantly across both study stratifications. Males were much more prevalent in the rapid progression group (53.4% vs. 31.9%) and among hypertensive patients (55.0% vs. 45.0%). Additionally, hypertensive patients were significantly older and had higher BMI compared to non-hypertensive individuals. Regarding genetic profiles, the distribution of *PKD* mutations differed significantly by disease progression: *PKD1* mutations were markedly more frequent in the rapid progression group (71.0%) compared to the slow progression group (50.0%). No significant difference in mutation distribution was identified between hypertensive and non-hypertensive patients. In terms of biochemical parameters, patients with rapid progression exhibited significantly higher plasma creatinine, uric acid, triglycerides, hemoglobin, and proteinuria, and significantly lower GFR and HDL-C levels. A similar pattern of metabolic and renal impairment was observed in the hypertension analysis: hypertensive patients displayed elevated creatinine, uric acid, triglycerides, total cholesterol, LDL-C, CRP, and proteinuria, with a concomitant reduction in GFR, albumin, and HDL-C.

### 3.1. Comparison of Amino Acid Levels in Progression Status and Hypertension Status

**Figure 1:**
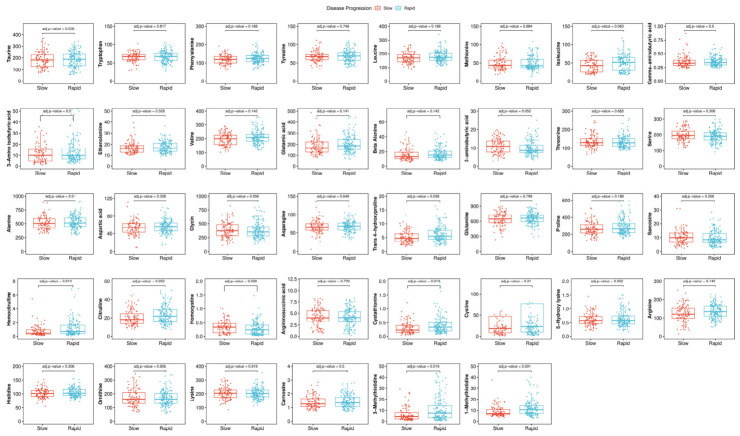
Box plots comparing amino acid levels between slow and rapid progression statuses, defined according to the Progression Status classification. Each panel shows the distribution of a specific amino acid, with slow progression on the left and rapid progression on the right. Horizontal lines within the boxes indicate median values, box edges represent the interquartile range (IQR), and whiskers extend to 1.5 × IQR. Outliers are displayed as individual points. Adjusted *p*-values were calculated using the Wilcoxon rank-sum test and adjusted for multiple comparisons using the Benjamini–Hochberg false discovery rate (FDR) correction; adjusted *p*-values are reported for each amino acid.

**Figure 2:**
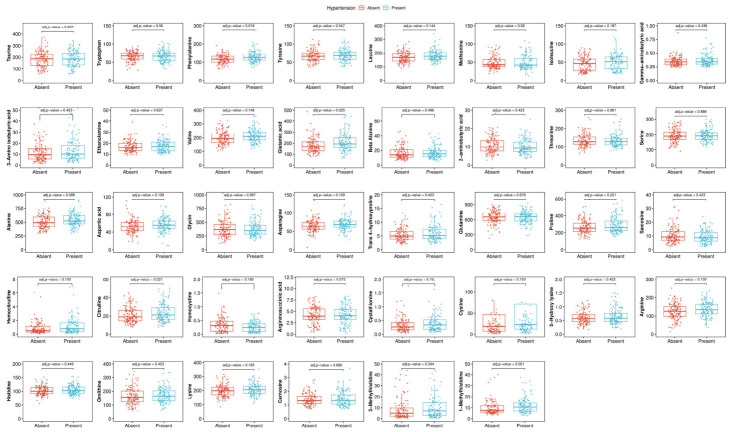
Box plots comparing amino acid levels between participants with and without hypertension. Each panel displays the distribution of a specific amino acid, with the non-hypertensive group shown on the left and the hypertensive group on the right. Horizontal lines within the boxes indicate median values, box edges represent the interquartile range (IQR), and whiskers extend to 1.5 × IQR. Outliers are displayed as individual points. Adjusted *p*-values were calculated using the Wilcoxon rank-sum test and adjusted for multiple comparisons using the Benjamini–Hochberg false discovery rate (FDR) correction; adjusted *p*-values are reported for each amino acid.

Targeted metabolomics analysis was performed to quantify 38 plasma amino acids. The concentrations of these amino acids were compared between the disease progression groups (rapid vs. slow) (Figure 1, Table S1) and hypertension status (present vs. absent) (Figure 2, Table S2).

#### 3.1.1. Progression Status

The comparison of amino acid levels between the progression groups is detailed in Figure 1 and Table S1. Levels of isoleucine, valine, glutamic acid, beta alanine, trans-4-hydroxyproline, homocitrulline, citrulline, cystathionine, 3-methylhistidine, and 1-methylhistidine were significantly higher in the rapid progression group compared to the slow progression group. In contrast, homocysteine and 2-aminobutyric acid levels were significantly higher in the slow- progression-group. Other amino acid levels did not show statistically significant differences between the two groups.

#### 3.1.2. Hypertension Status

Metabolic profiling also revealed alterations in amino acid concentrations associated with hypertension status (Figure 2 and Table S2). Levels of phenylalanine, leucine, valine, glutamic acid, alanine, aspartic acid, asparagine, homocitrulline, cystine, arginine, and 1-methylhistidine were significantly higher in hypertensive patients compared to the non-hypertensive group. Other amino acids did not show significant differences between the two groups.

### 3.2. Prognostic Implications and Correlation Analysis

#### 3.2.1. Survival Analysis

**Figure 3:**
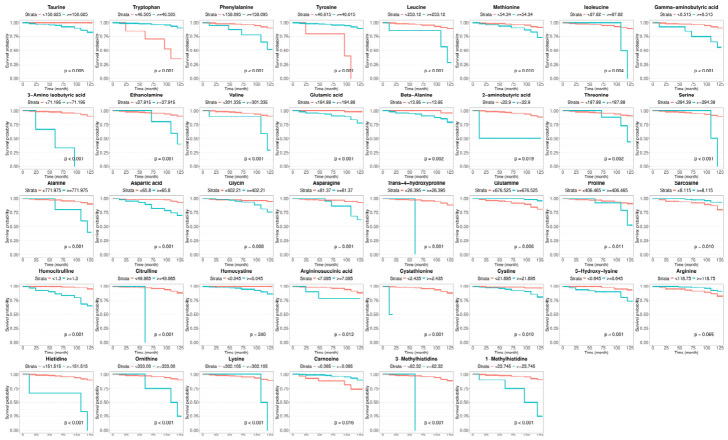
Kaplan–Meier survival curves for each amino acid, stratified by optimal cut-off values determined via the log-rank test. Each panel represents one amino acid, with survival probability plotted against time (months). The blue line corresponds to patients with amino acid concentrations below the cut-off value, and the red line corresponds to those above the cut-off. Cut-off values and log-rank *p*-values are indicated for each plot.

To further explore the prognostic implications of these metabolic changes, we conducted survival analyses for each amino acid. Patients were dichotomized into high- and low-level groups based on optimal cut-off values for each amino acid. The Kaplan–Meier plots in Figure 3 illustrate the association between amino acid levels and patient survival. Cut-off values were determined using the log-rank test statistics, and the selected amino acid thresholds successfully distinguished differences in survival probability.

Amino acid levels demonstrated distinct survival trajectories, except for homocysteine and arginine. All other evaluated amino acids showed statistically significant associations with survival. For these significant amino acids, survival probability decreased over time when their levels exceeded the determined cut-off values. In particular, abrupt decreases in survival were observed for isoleucine (cut-off > 87.82), 3-amino isobutyric acid (cut-off > 71.20), 2-aminobutyric acid (cut-off > 22.90), serine (cut-off > 294.39), trans-4-hydroxyproline (cut-off > 26.40), citrulline (cut-off > 49.99), cystathionine (cut-off > 2.44), lysine (cut-off > 302.11), and 3-methylhistidine (cut-off > 62.32).

#### 3.2.2. Correlation with Clinical Parameters

**Figure 4:**
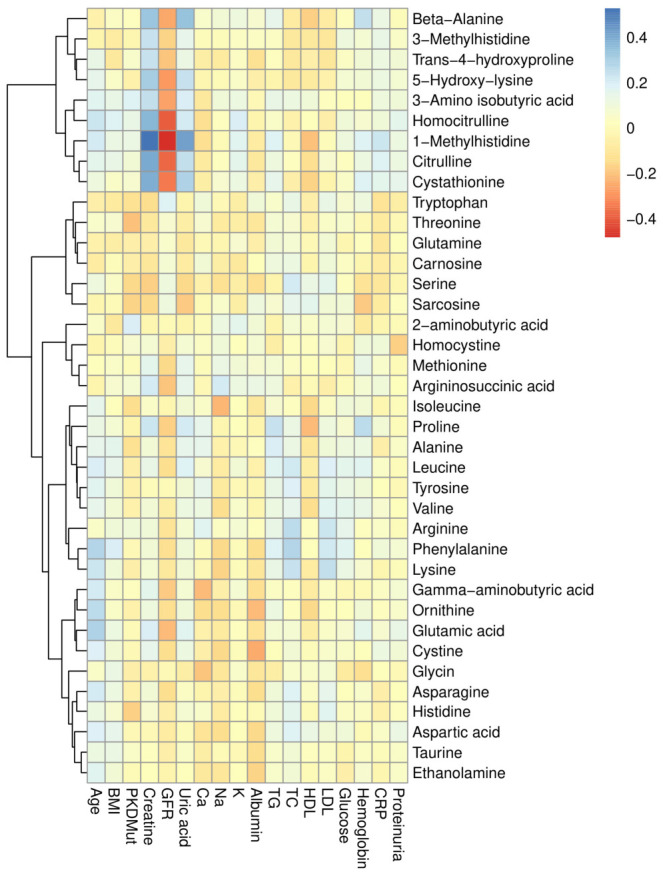
The heatmap showed 38 amino acids and 18 clinical parameters. The calculated Spearman correlation coefficients are visualized using a heatmap. Rows represent 38 amino acids, and columns represent 18 clinical parameters. Each cell is color-coded according to the corresponding amino acid and laboratory measurement correlation coefficient. The dendrogram displays the clustering of amino acids based on their correlation coefficients.

Spearman correlation analysis was performed to investigate the relationship between the 38 amino acid concentrations and 18 clinical parameters. A heatmap visualizing the calculated correlation coefficients is presented in Figure 4. This analysis identified a distinct cluster of amino acids strongly associated with renal function markers. The levels of 1-methylhistidine, citrulline, cystathionine, trans-4-hydroxyproline, 5-hydroxy-lysine, 3-amino isobutyric acid, and homocitrulline were positively correlated with creatinine and uric acid parameters. Furthermore, the same group of metabolites was negatively correlated with GFR.

### 3.3. Hierarchical Clustering Analysis

To visualize the amino acid profiles across the entire cohort, the concentrations of the 38 measured amino acids for all 254 samples are represented in a heatmap (Figure S1). In this plot, individual samples are organized in rows and amino acids in columns, with cells colored according to measured concentrations. The generated dendrogram shows clustering of samples based on similarities in their overall amino acid levels, providing a comprehensive view of the metabolic relationships between individual patients within progression status, hypertension, and mortality.

### 3.4. Pathway Analysis

Pathway enrichment analysis revealed significant metabolic alterations in ADPKD patients, with distinct profiles associated with disease progression and hypertension (Table 2). Regarding disease progression, the most impacted pathways were valine, leucine, and isoleucine biosynthesis (*p* = 0.000782), arginine biosynthesis (*p* = 0.002497), and histidine metabolism (*p* = 0.003274). In patients with hypertension, the most significantly altered pathways were alanine, aspartate, and glutamate metabolism (*p* = 5.12 × 10^−6^), arginine biosynthesis (*p* = 2.96 × 10^−5^), and valine, leucine, and isoleucine biosynthesis (*p* = 0.00061).

### 3.5. Performance of Machine Learning Prediction Models

For both disease progression and hypertension, models constructed using the combined set of clinical and amino acid variables achieved higher predictive performance than those based on either clinical or amino acid variables alone.

#### 3.5.1. Disease Progression Prediction

**Figure 5:**
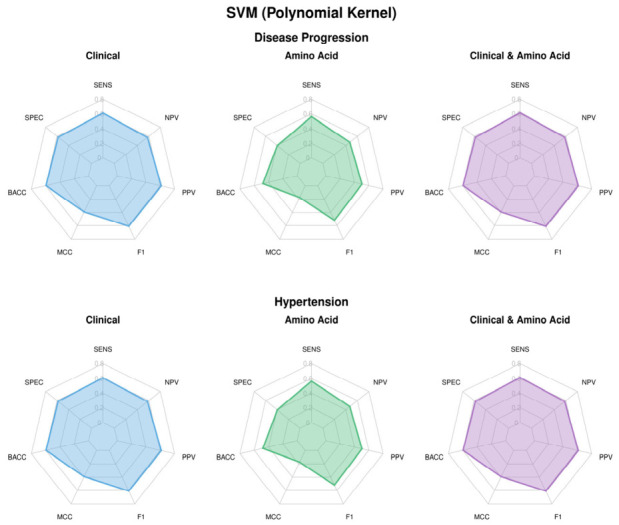
Radar plots illustrating the comparative performance of disease progression and hypertension prediction models based on an SVM (polynomial kernel) algorithm using the Lasso feature selection method.

**Figure 6:**
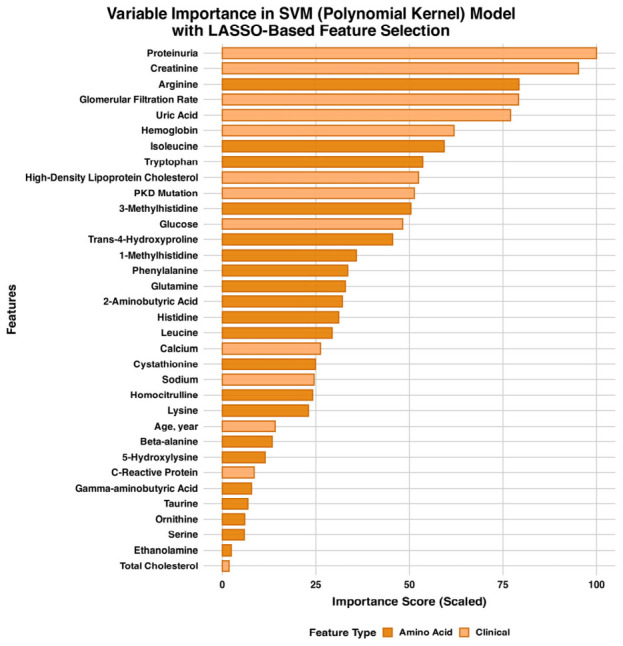
Variable importance of Lasso-selected clinical and amino acid features in SVM (polynomial kernel) for disease progression prediction.

The model based solely on clinical variables yielded a balanced accuracy (BACC) of 0.556, while the model based solely on amino acid variables achieved a BACC of 0.541. When clinical and amino acid variables were combined, model performance improved, with BACC increasing to 0.593. In addition, the combined model demonstrated higher specificity, MCC, and PPV compared with the single-feature-set models (Figure 5, Table S3). Variable importance analysis of the combined disease progression model indicated that the most influential clinical variables were proteinuria, creatinine, GFR, uric acid, and hemoglobin. The top contributing amino acids were arginine, isoleucine, tryptophan, 3-methylhistidine, and trans-4-hydroxyproline (Figure 6).

#### 3.5.2. Hypertension Prediction

**Figure 7:**
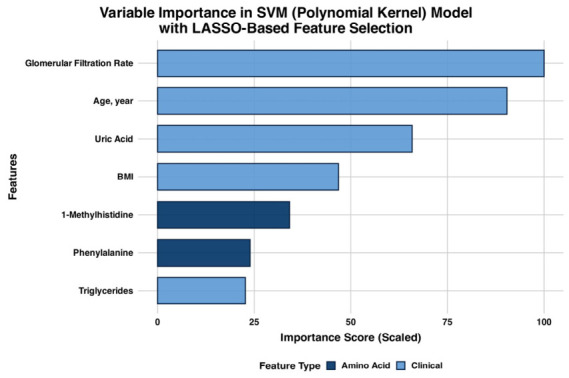
Variable importance of Lasso-selected clinical and amino acid features in SVM (polynomial kernel) for hypertension prediction.

The model using clinical variables alone achieved a BACC of 0.745, whereas the amino acid-only model performed worse (BACC = 0.600). The combined clinical and amino acid model slightly improved performance, yielding a BACC of 0.746. Furthermore, the combined model showed higher sensitivity, MCC, F1-score, and NPV compared with the individual models (Figure 5, Table S3). For hypertension, the most important clinical predictors were GFR, age, uric acid, body mass index (BMI), and triglyceride levels. Among amino acids, 1-methylhistidine and phenylalanine were identified as the strongest contributors to the model (Figure 7). Overall, integrating amino acid profiles with clinical variables led to minimal improvement in hypertension prediction and relatively low performance in disease progression prediction, indicating that the additional contribution of multi-domain data integration is limited.

## 4. Discussion

In this study, we implemented a targeted metabolomic analysis that was integrated with machine learning to unravel plasma amino acid signatures associated with ADPKD progression. Our findings indicated that specific amino acids—including BCAAs (isoleucine and valine), glutamic acid, and methylhistidines─ were elevated in rapid progression compared to slow progression. Similarly, the hypertension exhibited elevated BCAAs (leucine, valine), glutamic acid, asparagine, arginine, and 1-methylhistidine. By leveraging machine learning, we demonstrated that integrating these amino acid profiles with clinical data yielded a modest incremental value over clinical models alone in predictive accuracy for disease progression and hypertension. Specifically, clinical parameters, proteinuria, creatinine, GFR, uric acid, and amino acids; arginine, isoleucine, tryptophan, and methylhistidine were the top predictors of ADPKD progression in SVM-polynomial models. For hypertension, GFR, age, uric acid, and BMI, alongside 1-methylhistidine and phenylalanine, emerged as the most significant predictors.

The increased levels of BCAAs in the rapid progression and hypertension groups validate findings from our previous untargeted metabolomics study of ADPKD patients [14], identifying a role for BCAAs and dysregulation of the valine, leucine, and isoleucine biosynthesis pathway. These results are consistent with prior experimental and clinical findings, implicating BCAA metabolism in ADPKD pathogenesis [8,13], where BCAAs—particularly leucine—act as established activators of the mTOR signaling pathway, driving cyst epithelial cell proliferation and expansion [9].

Furthermore, the increased levels of asparagine and its precursor aspartic acid, along with arginine, further validate our previous untargeted metabolomics findings [14]. These amino acids were significantly accumulated in the hypertensive ADPKD group and are integral to arginine biosynthesis and to the alanine, aspartate, and glutamate metabolism pathways, which were enriched in our analysis. The elevation of asparagine reflects its central role in sustaining protein synthesis and the metabolic demands of proliferating PKD cells, consistent with previous in vitro, in vivo, and pediatric cohort findings [8,12,24]. Moreover, the high arginine abundance is implicated in both CKD and ADPKD, which in turn affects nitric oxide (NO) synthesis. This accumulation has the potential to promote a state of arginine auxotrophy, where reduced expression of argininosuccinate synthetase 1 (ASS1). ASS1 is the enzyme responsible for converting citrulline into arginosuccinate, the immediate precursor to arginine, rendering cells dependent on external arginine to sustain the proliferation that leads to cystogenesis. [25,26]. Simultaneously, the elevated levels of citrulline and homocitrulline observed in the rapid progression group reflect this disruption in the arginine-NO pathway, which is likely to contribute to endothelial dysfunction and vascular maintenance [24].

We identified significant alterations in histidine-related metabolites and glutamic acid, supporting a broader framework of amino acid-driven metabolic reprogramming in ADPKD [13,27]. The significant elevation of methylhistidines highlights altered histidine metabolism as a top-enriched pathway. While these metabolites accumulate as uremic retention solutes due to declining GFR, 3-methylhistidine offers distinct physiological insights as a specific biomarker for myofibrillar protein degradation and endogenous muscle turnover [28,29]. Mechanistically, histidine derivatives can contribute to glutamate production, fueling glutaminolysis to replenish the tricarboxylic acid (TCA) cycle in proliferating cyst epithelial cells [26,27]. Consequently, the elevated glutamate levels observed in rapidly progressing and hypertensive patients, consistent with the “Warburg effect,” support the anabolic and energetic demands of cyst expansion [10,26]. These alterations are likely attributed not only to reduced renal clearance but also to the disruption of the kidney’s intrinsic role in amino acid interconversion and nitrogen handling [11].

Furthermore, tryptophan, one of the most influential amino acid-related features in our SVM-Polynomial models, is often altered in inflammatory conditions. Its dysregulation is linked to elevated markers, such as C-reactive protein (CRP), which was significantly higher in our hypertensive patients [30]. Tryptophan serves as a precursor for the kynurenine pathway, which is known to be altered in ADPKD and to correlate with inflammatory cytokines [31,32].

In addition, we observed associations between specific amino acids and renal function markers: 1-methylhistidine, citrulline, cystathionine, trans-4-hydroxyproline, 5-hydroxy-lysine, 3-amino isobutyric acid, and homocitrulline were positively correlated with creatinine and uric acid and negatively correlated with GFR. These associations suggest that their accumulation is a direct consequence of reduced renal clearance, characterizing them as putative uremic retention solutes. Specifically, 1-methylhistidine (a muscle-derived metabolite from dietary sources) and 3-aminoisobutanoic acid (a thymine degradation product) are established uremic solutes that accumulate as GFR declines [33]. Similarly, the elevation of homocitrulline, a product of protein carbamylation, results from a high nitrogenous waste burden; high urea levels lead to increased cyanate, which carbamylates lysine to form homocitrulline [34].

Negative correlations of these amino acids with GFR confirm their retention as solutes even in early-stage renal decline, suggesting complex mechanisms beyond simple filtration failure. The kidney plays a key role in amino acid metabolism (synthesis and degradation). For example, the kidney extracts citrulline to synthesize arginine [35]. Renal injury inhibits this enzymatic conversion (via ASS and ASL), leading to elevated circulating citrulline, which may contribute to endothelial dysfunction and CKD progression [36]. Furthermore, elevated levels of trans-4-hydroxyproline and 5-hydroxylysine, derived exclusively from collagen degradation, correlate with renal fibrosis, reflecting the kidney’s failure to filter these breakdown products [33,37]. Additionally, patients with rapid progression and hypertension demonstrated significantly higher levels of proteinuria, which exhibited the highest importance score in our SVM-Polynomial models. Proteinuria is a robust biomarker of ADPKD severity; its strong association with disease progression in our models suggests it serves as a clinical indicator alongside metabolic reprogramming. The link between proteinuria and enhanced glycolytic markers suggests renal injury occurs concurrently with systemic metabolic shifts [38].

Our survival analysis indicated that isoleucine, 3-amino isobutyric acid, trans-4-hydroxyproline, citrulline, and 3-methylhistidine emerged as critical determinants of patient survival, with patients exceeding specific thresholds facing significantly worse outcomes. Based on these findings, we propose that elevated levels of BCAAs, arginine, asparagine, and methylhistidine, combined with clinical parameters, may serve as a prognostic biomarker for predicting ADPKD progression.

The primary strengths of this study lie in its longitudinal design, the relatively large sample size for a rare disease cohort, and the integration of robust machine learning techniques to predict clinical outcomes. Nevertheless, several limitations of this study should be recognized. First, the single-center setting may restrict our ability to generalize these metabolic changes to broader populations with different genetic or environmental backgrounds. To address this, future multi-center studies are required to confirm the potential of these metabolic changes as early prognostic biomarkers. Second, the study benefited from long-term clinical follow-up, allowing for survival analysis. However, metabolomic profiling was performed at a single baseline time point. As a result, we were unable to evaluate how longitudinal trajectories or temporal fluctuations in amino acid profiles correlate with the dynamic decline in renal function. Third, although we identified strong correlations with clinical parameters, the influence of unmeasured dietary intake cannot be entirely excluded. Furthermore, plasma amino acid concentrations may be substantially influenced by non-disease-related factors such as fasting status, low-protein or ketogenic diets, amino acid supplementation, and fluctuations in insulin resistance. Additionally, concomitant medications, including tolvaptan, RAAS inhibitors, diuretics, and SGLT2 inhibitors, may act as confounders. The lack of comprehensive data on these variables is a limitation of this study, and future research should incorporate these factors to isolate disease-specific metabolic shifts better. Fourth, the modest improvement in predictive accuracy provided by the combined models is likely attributable to the high collinearity between specific altered amino acids and established clinical markers such as GFR. Consequently, while these metabolites reflect renal decline, they contribute limited independent predictive power to the machine learning models. Furthermore, the inherent biological variance in plasma amino acids driven by unmeasured dietary and environmental factors can introduce statistical noise. Therefore, given the time-consuming and costly nature of targeted LC-MS/MS, this slight predictive gain does not currently justify its routine clinical application for prognostication alone. Instead, the primary value of these findings lies in revealing the underlying pathophysiological mechanisms of ADPKD as an exploratory tool. Finally, as an independent external validation cohort was not available, the prognostic thresholds and machine learning performances identified in our exploratory analysis require confirmation in future studies.

## 5. Conclusions

ADPKD is the most prevalent inherited kidney disorder, characterized by clinical heterogeneity and different clinical complications, such as hypertension, which complicate disease management. While the progression of ADPKD can be monitored using imaging-based tools or blood tests, functional decline is frequently captured late. Consequently, there is a critical need for biomarkers to enable early detection of ADPKD progression and to support personalized interventions. In this context, our targeted metabolomics analysis offers compelling evidence that specific disruptions in plasma amino acid metabolism, particularly in branched-chain amino acids (valine, leucine, isoleucine), glutamic acid, methylhistidines, and metabolites of the arginine-NO pathway (citrulline, homocitrulline), are closely linked to disease progression and hypertension.

By leveraging machine learning, we have shown that integrating these metabolic signatures with clinical indicators such as proteinuria, creatinine, uric acid, and GFR significantly provides incremental improvement in predictive performance compared to models using clinical data alone. These findings suggest that targeted amino acid profiling can complement existing risk-stratification approaches, providing more profound biological insights into the metabolic reprogramming driving cystogenesis. Ultimately, these biomarkers offer potential utility for monitoring therapeutic response and enabling more timely, personalized interventions for patients with ADPKD.

## Figures and Tables

**Table 1:** Demographic and laboratory characteristics of study groups.

Variable	Progression Status		*p*-Value	Hypertension Status		*p*-Value
	**Slow (*n* = 72)**	**Rapid (*n* = 131)**		**Absent (*n* = 92)**	**Present (*n* = 111)**	
Age, year	46.60 ± 14.63	47.69 ± 13.09	0.584	40.20 ± 11.38	53.20 ± 12.51	**<0.001**
Gender						
Male	23 (31.9)	70 (53.4)	**0.003**	32 (34.8)	61 (55.0)	**0.004**
Female	49 (68.1)	61 (46.6)		60 (65.2)	50 (45.0)	
BMI *	25.00 (23.13–27.00)	25.00 (23.00–28.00)	0.775	24.00 (21.00–27.00)	26.00 (24.00–28.00)	**<0.001**
PKD * mutation						
*PKD1*	36 (50.0) ^1^	93 (71.0) ^2^	**0.002**	61 (66.3)	68 (61.3)	0.175
*PKD2*	23 (31.9) ^1^	16 (12.2) ^2^		20 (21.7)	19 (17.1)	
*PKD3*	13 (18.1) ^1^	22 (16.8) ^1^		11 (12.0)	24 (21.6)	
Creatinine	0.77 (0.63–1.00)	1.09 (0.80–1.56)	**<0.001**	0.75 (0.65–0.95)	1.20 (0.81–1.60)	**<0.001**
GFR	106.00 (80.50–117.58)	76.00 (46.00–105.00)	**<0.001**	107.00 (88.50–119.90)	66.00 (37.40–93)	**<0.001**
Uric acid	4.69 (3.83–5.68)	5.80 (4.40–7.10)	**<0.001**	4.64 (3.53–5.70)	6.00 (4.80–7.10)	**<0.001**
Calcium	9.40 (9.03–9.60)	9.40 (9.10–9.70)	0.363	9.40 (9.10–9.60)	9.40 (9.00–9.70)	0.803
Sodium	140.00 (139.00–141.75)	140.00 (138.00–141.00)	0.460	140.00 (139.00–142.00)	140.00 (138.00–141.00)	0.515
Potassium	4.30 (4.00–4.50)	4.30 (4.10–4.60)	0.249	4.30 (4.10–4.50)	4.30 (4.00–4.60)	0.939
Albumin	4.50 (4.10–4.70)	4.30 (4.00–4.70)	0.461	4.50 (4.20–4.74)	4.20 (4.00–4.50)	**<0.001**
Triglycerides	103.00 (77.25–165.00)	130.00(98.00–175.00)	**0.013**	101.50(78.25–142.50)	134.00(103.00–192.00)	**<0.001**
Total Cholesterol	190.50 (161.00–209.50)	191.00 (158.00–210.00)	0.709	181.50 (149.25–202.75)	193.00 (167.00–214.00)	**0.004**
HDL	45.50 (37.00–61.25)	42.00 (36.00–51.00)	**0.031**	45.50 (39.25–58.00)	42.00 (35.00–50.00)	**0.012**
LDL	109.00 (87.75–135.75)	112.00 (86.00–134.00)	0.898	104.00 (79.25–125.00)	122.00 (95.00–142.00)	**0.001**
Glucose	90.00 (83.25–97.25)	92.00 (85.00–102.00)	0.246	90.00 (85.00–95.00)	93.00 (85.00–105.00)	0.094
Hemoglobin	13.30 (12.63–15.00)	14.30 (13.00–15.50)	**0.014**	13.40 (12.80–15.08)	14.50 (12.90–15.40)	0.070
CRP	3.00 (2.10–4.58)	3.00 (2.00–5.00)	0.725	3.00 (1.10–4.29)	3.00 (3.00–5.00)	**0.003**
Proteinuria	0.10 (0.07–0.15)	0.14 (0.10–0.27)	**<0.001**	0.10 (0.08–0.15)	0.15 (0.10–0.30)	**0.001**

* BMI: Body mass index; * *PKD*: polycystic kidney disease. The values are demonstrated as mean ± standard deviation, median (25th*–*75th percentile), and n (%). Different lowercase letters (1, 2, and 3) in the same row indicate a statistically significant difference between groups. The statistically significant values were marked in bold.

**Table 2:** Pathway enrichment analysis for progression and hypertension status.

Pathways	Total	Expected	Hits	Raw *p-*Value	−log10 (*p-*Value)	FDR	Impact
Valine, leucine, and isoleucine biosynthesis	8	0.045226	2	0.000782	3.1068	0.062561	0
Arginine biosynthesis	14	0.079146	2	0.002497	2.6026	0.087294	0.3617
Histidine metabolism	16	0.090452	2	0.003274	2.485	0.087294	0
Pantothenate and CoA biosynthesis	20	0.11307	2	0.005122	2.2905	0.10245	0.04762
Cysteine and methionine metabolism	33	0.18656	2	0.0137	1.8633	0.21604	0.22033
Arginine and proline metabolism	36	0.20352	2	0.016203	1.7904	0.21604	0.02093
Valine, leucine, and isoleucine degradation	40	0.22613	2	0.019825	1.7028	0.22657	0
Nitrogen metabolism	6	0.03392	1	0.033496	1.475	0.33496	0
Butanoate metabolism	15	0.084799	1	0.081871	1.0869	0.72774	0
beta-Alanine metabolism	21	0.11872	1	0.11291	0.94726	0.81041	0.39925
Propanoate metabolism	22	0.12437	1	0.11799	0.92814	0.81041	0
One carbon pool by folate	26	0.14698	1	0.13806	0.85992	0.81041	0.04489
Glutathione metabolism	28	0.15829	1	0.14795	0.82989	0.81041	0.01966
Alanine, aspartate, and glutamate metabolism	28	0.15829	1	0.14795	0.82989	0.81041	0.19712
Porphyrin metabolism	31	0.17525	1	0.16258	0.78893	0.81041	0

## Data Availability

The datasets used and/or analyzed in the current study are available from the corresponding author.

## Supplementary Materials

The following supporting information can be downloaded at: https://www.mdpi.com/article/10.3390/jcm15145340/s1, Table S1: Comparison of amino acid levels between disease progression groups; Table S2: Comparison of amino acid levels between hypertension groups; Table S3: Performance metrics for disease progression and hypertension prediction using the SVM (polynomial kernel) model with Lasso feature selection; Figure S1: The heatmap shows the concentration of 38 amino acids in 254 samples. There are 254 samples per row and 38 amino acids per column. Each cell is colored according to the sample’s corresponding amino acid measurement. The dendrogram shows the samples grouped by amino acid level.

## Abbreviations

The following abbreviations are used in this manuscript:

ADPKD	Autosomal Dominant Polycystic Kidney Disease
ESRD	End-Stage Renal Disease
SVM	Support Vector Machine
GFR	Glomerular Filtration Rate
BCAA	Branched-Chain Amino Acid
CKD	Chronic Kidney Disease
BMI	Body Mass Index
MIC	Mayo Imaging Classification
