# Supplementary material for: Plasma Amino Acid Signatures Associated with Disease Progression and Hypertension in Autosomal Dominant Polycystic Kidney Disease: A Targeted Metabolomics and Machine Learning Approach
Source: J Clin Med. 2026 Jul 8;15(14):5340. doi: 10.3390/jcm15145340 (PMC13410093; doi:10.3390/jcm15145340)
Supplement: Supplementary file 1 [file jcm-15-05340-s001.zip › jcm-4264727-supplementary.pdf]

Table S1. Comparison of amino acid levels between disease progression groups

Amino Acid	Disease Progression		Total	adj. *p*-value*
	**Slow (*n*=72)**	**Rapid (*n*=131)**		
Taurine	182.28(126.82-229.02)	188.39(134.34-235.38)	186.18(132.06-232.73)	0.502
Tryptophan	66.82(58.21-73.91)	67.21(55.85-76.65)	67.06(56.85-75.35)	0.731
Phenylalanine	118.47(99.81-134.38)	124.09(107.20-139.81)	122.98(104.57-137.75)	0.079
Tyrosine	66.58(58.38-73.84)	68.38(57.16-78.04)	67.72(57.38-76.87)	0.611
Leucine	168.73(142.00-194.71)	173.15(152.90-205.46)	172.00(149.27-200.87)	0.070
Methionine	43.83(35.94-57.47)	42.70(37.19-59.08)	43.00(36.97-57.92)	0.814
Isoleucine	43.04(25.38-56.96)	51.66(29.98-64.03)	49.30(29.12-62.25)	**0.015**
Gamma aminobutyric acid	0.33(0.29-0.38)	0.34(0.30-0.40)	0.34(0.29-0.40)	0.337
3-Amino isobutyric acid	10.14(4.83-15.52)	10.04(6.78-16.52)	10.11(6.23-15.97)	0.306
Ethanolamine	16.24(12.85-18.91)	16.81(13.82-21.02)	16.64(13.25-20.31)	0.190
Valine	199.40(158.95-224.58)	207.79(182.79-232.65)	204.05(172.70-232.07)	**0.042**
Glutamic acid	168.54(130.18-221.70)	187.46(154.42-242.30)	183.50(146.88-235.51)	**0.037**
Beta Alanine	13.38(10.44-19.26)	15.45(12.03-20.71)	14.88(11.52-20.29)	**0.045**
2-aminobutyric acid	10.96(7.82-13.78)	8.71(7.59-11.56)	9.40(7.66-12.98)	**0.011**
Threonine	129.52(114.70-150.15)	128.72(109.76-151.17)	128.95(112.97-151.01)	0.525
Serine	194.03(174.58-223.62)	189.95(167.66-213.98)	191.88(168.34-216.50)	0.153
Alanine	504.36(418.74-591.82)	512.18(446.66-606.53)	510.08(437.93-603.97)	0.341
Aspartic acid	53.48(42.90-62.80)	55.82(45.91-64.03)	54.71(45.36-63.08)	0.183
Glycin	369.41(282.20-483.49)	348.95(278.60-443.42)	362.87(280.12-459.15)	0.395
Asparagine	65.55(56.80-74.93)	68.16(57.68-76.84)	66.82(57.53-76.23)	0.478
Trans-4-hydroxyproline	4.69(3.58-6.31)	5.44(4.36-7.51)	5.02(3.94-7.04)	**0.006**
Glutamine	647.70(570.07-727.01)	663.20(591.92-718.61)	657.34(578.68-724.83)	0.690
Proline	255.74(215.24-313.53)	262.03(219.11-327.54)	259.64(217.73-325.01)	0.077
Sarcosine	9.88(6.55-13.18)	8.46(6.40-12.79)	8.94(6.40-12.98)	0.146
Homocitrulline	0.51(0.32-0.98)	0.76(0.45-1.64)	0.68(0.37-1.33)	**0.001**
Citrulline	18.27(14.18-24.45)	21.80(16.04-28.90)	19.88(15.08-27.20)	**0.011**
Homocysteine	0.34(0.15-0.47)	0.24(0.10-0.41)	0.28(0.12-0.42)	**0.006**
Argininosuccinic acid	4.02(3.29-5.56)	4.06(3.19-5.35)	4.06(3.21-5.46)	0.694
Cystathionine	0.24(0.16-0.40)	0.34(0.21-0.50)	0.30(0.18-0.46)	**0.002**

Cystine	18.94(9.22-47.29)	23.58(10.81-76.83)	21.94(10.53-55.79)	0.163
5-Hydroxy lysine	0.58(0.46-0.72)	0.58(0.45-0.74)	0.58(0.45-0.74)	0.962
Arginine	120.43(97.84-151.64)	133.94(109.52-164.57)	129.37(102.41-160.80)	0.051
Histidine	100.97(90.71-111.18)	102.03(93.74-115.47)	101.47(91.99-114.07)	0.137
Ornithine	161.96(131.15-209.27)	161.86(130.94-201.75)	161.95(130.94-202.66)	0.931
Lysine	203.96(173.9-226.45)	202.09(178.07-226.32)	203.24(174.02-226.39)	0.871
Carnosine	1.27(1.07-1.65)	1.36(1.09-1.76)	1.33(1.08-1.72)	0.342
3-Methylhistidine	4.14(2.04-8.14)	7.60(3.11-14.38)	5.01(2.66-12.31)	**0.002**
1-Methylhistidine	7.23(5.61-10.95)	10.61(7.02-14.42)	8.78(6.21-12.96)	**<0.001**

*adj.*p*-value: *p*-value adjusted for multiple comparisons using the Benjamini–Hochberg method. The statistically significant values were marked in bold.

Table S2. Comparison of amino acid levels between hypertension groups

**Amino Acid**	**Hypertension**		**Total**	**adj. *p*-value***
	**Absent (*n*=92)**	**Present (*n*=111)**		
Taurine	186.35(130.67-228.70)	185.08(134.17-235.51)	185.90(131.95-232.19)	0.536
Tryptophan	67.38(58.66-75.16)	66.79(55.33-75.45)	67.05(56.84-75.29)	0.442
Phenylalanine	116.84(100.35-133.20)	126.13(111.78-142.85)	122.88(104.25-137.66)	**0.001**
Tyrosine	66.41(56.83-75.27)	68.49(58.18-77.85)	67.67(57.34-76.68)	0.403
Leucine	168.60(142.00-197.50)	177.08(154.92-203.50)	171.47(149.26-201.00)	**0.034**
Methionine	43.63(38.10-57.38)	42.34(35.36-59.81)	43.00(36.97-58.02)	0.439
Isoleucine	46.40(27.34-58.51)	51.44(31.70-64.24)	49.21(29.09-62.29)	0.074
Gamma aminobutyric acid	0.34(0.29-0.39)	0.34(0.30-0.40)	0.34(0.29-0.40)	0.287
3-Amino isobutyric acid	9.61(5.91-14.91)	10.18(6.58-18.04)	10.12(6.28-16.14)	0.246
Ethanolamine	16.12(13.36-19.94)	17.20(13.14-20.99)	16.64(13.23-20.31)	0.534
Valine	194.95(165.73-227.32)	209.93(184.08-240.05)	204.02(172.43-232.07)	**0.039**
Glutamic acid	167.39(132.84-212.18)	193.67(156.81-250.69)	183.07(145.92-235.73)	**0.002**
Beta Alanine	14.20(11.13-20.82)	15.47(11.82-20.20)	14.97(11.53-20.32)	0.345
2-aminobutyric acid	9.88(7.72-13.39)	9.23(7.40-12.64)	9.38(7.66-13.02)	0.229
Threonine	128.09(114.32-153.91)	129.47(111.33-147.46)	128.79(112.96-150.42)	0.770
Serine	190.54(167.84-217.80)	192.25(168.63-213.33)	191.72(168.27-216.04)	0.839
Alanine	492.08(418.10-591.88)	524.28(471.00-619.78)	510.15(437.83-604.02)	**0.009**
Aspartic acid	52.53(43.06-62.29)	56.03(48.28-65.43)	54.64(45.34-63.15)	**0.020**
Glycin	365.30(275.24-464.34)	349.11(282.16-448.40)	360.74(280.10-457.94)	0.997
Asparagine	64.25(55.64-74.04)	69.37(61.49-78.09)	66.81(57.48-76.38)	**0.019**
Trans-4-hydroxyproline	4.97(3.85-6.59)	5.12(4.04-7.44)	5.03(3.97-7.04)	0.258

Glutamine	653.36(585.7-728.93)	661.66(576.20-709.64)	656.89(578.46-723.42)	0.587
Proline	253.72(213.37-313.90)	262.26(224.78-338.84)	259.75(217.70-325.07)	0.094
Sarcosine	9.25(6.67-13.18)	8.70(6.04-12.65)	8.94(6.39-12.89)	0.243
Homocitrulline	0.56(0.37-1.16)	0.83(0.42-1.65)	0.68(0.38-1.34)	**0.023**
Citrulline	19.09(14.49-26.18)	21.2(15.83-29.02)	19.96(15.06-27.29)	0.099
Homocysteine	0.32(0.12-0.47)	0.25(0.11-0.38)	0.28(0.12-0.42)	0.065
Argininosuccini c acid	3.98(3.19-5.56)	4.07(3.23-5.36)	4.06(3.21-5.48)	0.947
Cystathionine	0.27(0.17-0.42)	0.34(0.19-0.52)	0.30(0.18-0.46)	0.057
Cystine	18.91(8.03-47.55)	22.83(12.78-72.12)	21.94(10.51-53.20)	**0.046**
5-Hydroxy lysine	0.57(0.45-0.72)	0.59(0.46-0.76)	0.58(0.45-0.74)	0.267
Arginine	125.90(98.39-151.59)	134.25(111.68-165.24)	129.29(102.25-160.05)	**0.023**
Histidine	100.12(91.37-112.40)	102.30(93.47-114.83)	101.39(91.98-113.99)	0.307
Ornithine	157.11(124.42-201.46)	165.64(132.27-202.84)	161.92(130.90-202.72)	0.225
Lysine	198.22(170.18-222.50)	205.57(180.55-228.62)	203.16(173.99-226.24)	0.068
Carnosine	1.33(1.11-1.65)	1.34(1.07-1.75)	1.33(1.08-1.72)	0.825
3-Methylhistidine	4.84(2.28-9.87)	7.17(2.93-14.71)	5.07(2.67-12.32)	0.163
1-Methylhistidine	7.77(5.77-11.91)	10.70(7.14-14.39)	8.81(6.23-13.00)	**<0.001**

*adj.*p*-value: *p*-value adjusted for multiple comparisons using the Benjamini–Hochberg method. The statistically significant values were marked in bold.

Table S3. **Performance metrics for disease progression and hypertension prediction using the SVM (polynomial kernel) model with Lasso feature selection.**

**Scenarios**	**Selected by Lasso**						
	**SENS* (95% CI*)**	**SPEC* (95% CI)**	**BACC* (95% CI)**	**MCC*(95% CI)**	**F1 Score (95% CI)**	**PPV* (95% CI)**	**NPV* (95% CI)**
**Disease Progression**							
**Clinical Data**	0.885(0.861-0.910)	0.226(0.191-0.261)	0.556(0.541-0.571)	0.162(0.121-0.202)	0.761(0.749-0.772)	0.672(0.658-0.686)	0.550(0.488-0.611)
**Amino Acid Data**	0.898(0.868-0.928)	0.184(0.147-0.222)	0.541(0.530-0.553)	0.153(0.120-0.186)	0.758(0.746-0.770)	0.663(0.648-0.678)	0.588(0.518-0.658)
**Clinical + Amino Acid Data**	0.825(0.798-0.853)	0.361(0.319-0.403)	0.593(0.576-0.611)	0.219(0.182-0.255)	0.751(0.738-0.765)	0.699(0.680-0.718)	0.557(0.513-0.601)
**Hypertension**							
**Clinical Data**	0.764(0.741-0.786)	0.726(0.707-0.745)	0.745(0.731-0.759)	0.489(0.461-0.517)	0.763(0.748-0.777)	0.768(0.751-0.786)	0.720(0.693-0.746)
**Amino Acid Data**	0.706(0.664-0.748)	0.495(0.441-0.549)	0.600(0.585-0.616)	0.216(0.186-0.247)	0.649(0.632-0.666)	0.632(0.604-0.659)	0.585(0.556-0.615)
**Clinical + Amino Acid Data**	0.769(0.746-0.792)	0.723(0.701-0.745)	0.746(0.729-0.763)	0.491(0.457-0.525)	0.765(0.748-0.782)	0.767(0.747-0.788)	0.723(0.696-0.750)

*SENS: Sensitivity; *SPEC: Specificity; *BACC: Balanced Accuracy; *MCC: Matthews Correlation Coefficient; *PPV: Positive Predictive Value; *NPV: Negative Predictive Value. *CI: Confidence Interval.

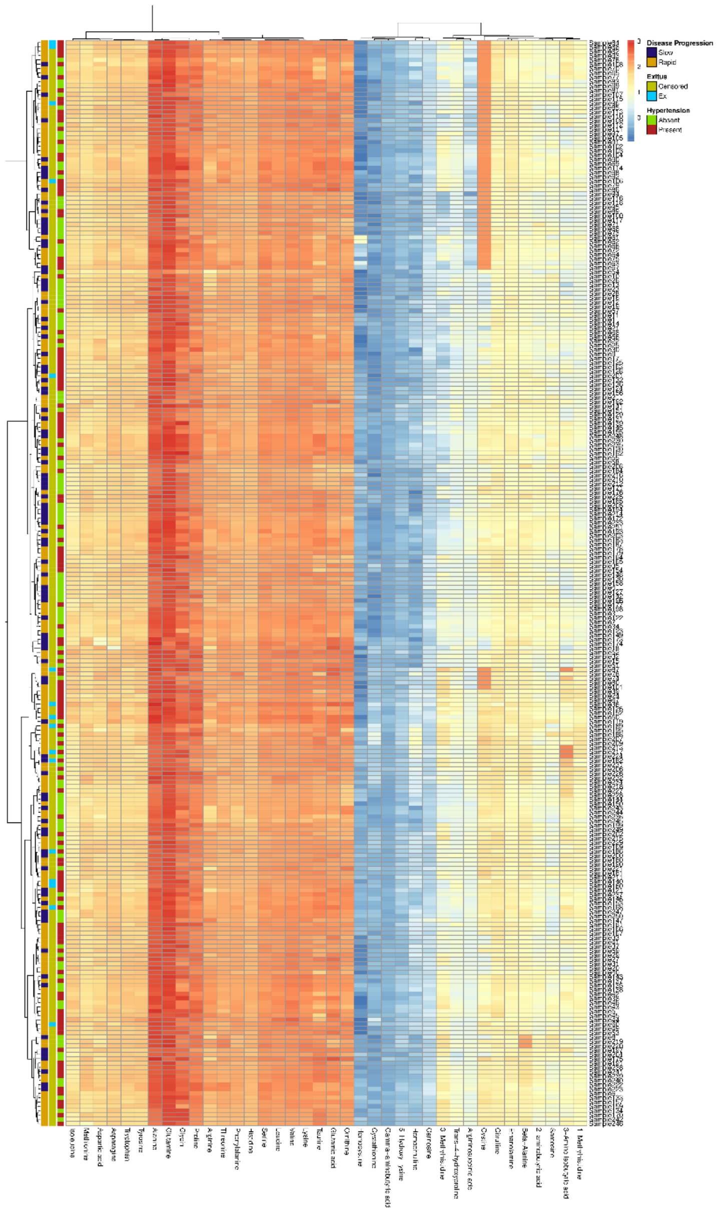
Disease Progression
Slow
Rapid
Exitus
Censored
Ex
Hypertension
Absent
Present

Figure S1. The heatmap shows the concentration of 38 amino acids in 254 samples. There are 254 samples per row and 38 amino acids per column. Each cell is colored according to the sample's corresponding amino acid measurement. The dendrogram shows the samples grouped by amino acid level.
